# Semantic encoding during language comprehension at single-cell resolution

**DOI:** 10.1038/s41586-024-07643-2

**Published:** 2024-07-03

**Authors:** Mohsen Jamali, Benjamin Grannan, Jing Cai, Arjun R. Khanna, William Muñoz, Irene Caprara, Angelique C. Paulk, Sydney S. Cash, Evelina Fedorenko, Ziv M. Williams

**Affiliations:** 1 Department of Neurosurgery, Massachusetts General Hospital, Harvard Medical School, Boston, MA USA; 2 Department of Neurology, Massachusetts General Hospital, Harvard Medical School, Boston, MA USA; 3 Center for Neurotechnology and Neurorecovery, Department of Neurology, Massachusetts General Hospital, Boston, MA USA; 4 Department of Brain and Cognitive Sciences and McGovern Institute for Brain Research, Massachusetts Institute of Technology, Cambridge, MA USA; 5 Harvard-MIT Division of Health Sciences and Technology, Boston, MA USA; 6 Harvard Medical School, Program in Neuroscience, Boston, MA USA

**Keywords:** Language, Neural decoding

## Abstract

From sequences of speech sounds^1,2^ or letters^3^, humans can extract rich and nuanced meaning through language. This capacity is essential for human communication. Yet, despite a growing understanding of the brain areas that support linguistic and semantic processing^4–12^, the derivation of linguistic meaning in neural tissue at the cellular level and over the timescale of action potentials remains largely unknown. Here we recorded from single cells in the left language-dominant prefrontal cortex as participants listened to semantically diverse sentences and naturalistic stories. By tracking their activities during natural speech processing, we discover a fine-scale cortical representation of semantic information by individual neurons. These neurons responded selectively to specific word meanings and reliably distinguished words from nonwords. Moreover, rather than responding to the words as fixed memory representations, their activities were highly dynamic, reflecting the words’ meanings based on their specific sentence contexts and independent of their phonetic form. Collectively, we show how these cell ensembles accurately predicted the broad semantic categories of the words as they were heard in real time during speech and how they tracked the sentences in which they appeared. We also show how they encoded the hierarchical structure of these meaning representations and how these representations mapped onto the cell population. Together, these findings reveal a finely detailed cortical organization of semantic representations at the neuron scale in humans and begin to illuminate the cellular-level processing of meaning during language comprehension.

## Abstract (web summary)

By tracking the activity of individual neurons using microarrays and Neuropixels probes, a study examines the representation of linguistic meaning, at the single-cell level, during natural speech processing in humans.

## Main

Humans are capable of communicating exceptionally detailed meanings through language. How neurons in the human brain represent linguistic meaning and what their functional organization may be, however, remain largely unknown. Initial perceptual processing of linguistic input is carried out by regions in the auditory cortex for speech^1,2^ or visual regions for reading^3^. From there, information flows to the amodal language-selective^9^ left-lateralized network of frontal and temporal regions that map word forms to word meanings and assemble them into phrase- and sentence-level representations^4,5,13^. Processing meanings extracted from language also engages widespread areas outside this language-selective network, with diverging evidence suggesting that semantic processing may be broadly distributed across the cortex^11^ or that it may alternatively be concentrated in a few semantic ‘hubs’ that process meaning from language as well as other modalities^7,12^. How linguistic and semantic information is represented at the basic computational level of individual neurons during natural language comprehension in humans, however, remains undefined.

 Despite a growing understanding of semantic processing from imaging studies, little is known about how neurons in humans process or represent word meanings during language comprehension. Further, although speech processing is strongly context dependent^14^, how contextual information influences meaning representations and how these changes may be instantiated within sentences at a cellular scale remain largely unknown. Finally, although our semantic knowledge is highly structured^15–17^, little is understood about how cells or cell ensembles represent the semantic relationships among words or word classes during speech processing and what their functional organization may be.

Single-neuronal recordings have the potential to begin unravelling some of the real-time dynamics of word and sentence comprehension at a combined spatial and temporal resolution that has largely been inaccessible through traditional human neuroscience approaches^18–20^. Here we used a rare opportunity to record from single cells in humans^18,19,21^ and begin investigating the moment-by-moment dynamics of natural language comprehension at the cellular scale.

## Single-neuron recordings during speech processing

**Fig. 1:**
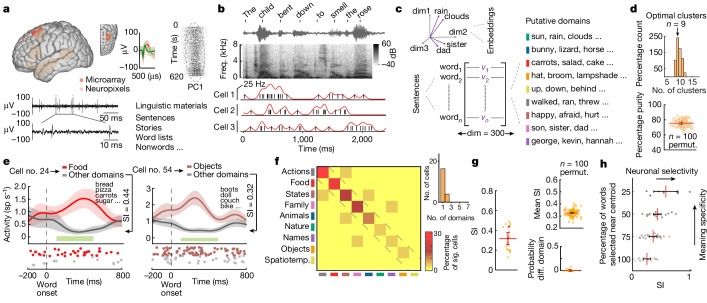
Semantic selectivity by single neurons during naturalistic speech processing. **a**, Left: single-neuron recordings were obtained from the left language-dominant prefrontal cortex. Recording locations for the microarray (red) and Neuropixels (beige) recordings (spm12; Extended Data Table 1) as well as an approximation of language-selective network areas (brown) are indicated. Right: the action potentials of putative neurons. **b**, Action potentials (black lines) and instantaneous firing rate (red trace) of each neuron were time-aligned to the onset of each word. Freq., frequency. **c**, Word embedding approach for identifying semantic domains. Here each word is represented as a 300-dimensional (dim) vector. **d**, Silhouette criterion analysis (upper) and purity measures (lower) characterized the separability and quality of the semantic domains (Extended Data Fig. 2a). permut., permutations. **e**, Peri-stimulus spike histograms (mean ± standard error of the mean (s.e.m.)) and rasters for two representative neurons. The horizontal green bars mark the window of analysis (100–500 ms from onset). sp, spikes. **f**, Left: confusion matrix illustrating the distribution of cells that exhibited selective responses to one or more semantic domains (*P* < 0.05, two-tailed rank-sum test, false discovery rate-adjusted). Spatiotemp., spatiotemporal.; sig. significant. Top right: numbers of cells that exhibited semantic selectivity. **g**, Left: SI of each neuron (*n* = 19) when compared across semantic domains. The SIs of two neurons are colour-coded to correspond to those shown in Fig. 1e. Upper right: mean SI across neurons when randomly selecting words from 60% of the sentences (mean SI = 0.33, CI = 0.32–0.33; across 100 iterations). Bottom right*:* probabilities of neurons exhibiting significant selectivity to their non-preferred semantic domains when randomly selecting words from 60% of the sentences (1.4 ± 0.5% mean ± s.e.m. different (diff.) domain). **h**, Relationship between increased meaning specificity (by decreasing the number of words on the basis of the words’ distance from each domain’s centroid) and response selectivity. The lines with error bars in **d**,**g**,**h** represent mean with 95% confidence limits.

**Extended Data Fig. 1:**
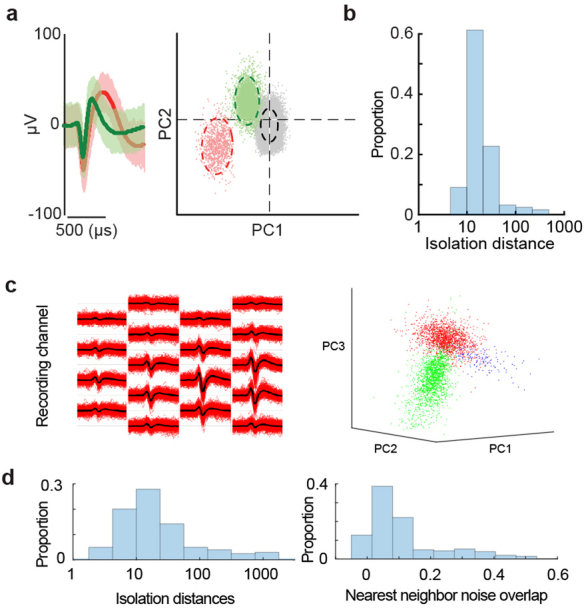
Language-related activity, recording stability, waveform morphology and isolation quality across recording techniques. **a**, Example of waveform morphologies displaying mean waveform ± 3 s.d and associated PC distributions used to isolate putative units from the tungsten microarray recordings. The horizontal bar indicates a 500 µs interval for scale. The gray areas in PC space represent noise. All single units recorded from the same electrode were required to display a high degree of separation in PC space. **b**, Isolation metrics of the single units obtained from the tungsten microarray recordings. **c**, *Left*, waveform morphologies observed across contacts in a Neuropixels array. *Right*, PC distributions used to isolate and cluster single units. **d**, Isolation distance and nearest neighbor noise overlap of the recorded units obtained from the Neuropixels arrays.

Single-neuronal recordings were obtained from the prefrontal cortex of the language-dominant hemisphere in a region centred along the left posterior middle frontal gyrus (Fig. 1a and Methods (‘Acute intraoperative single-neuronal recordings’) and Extended Data Fig. 1a). This region contains portions of the language-selective network together with several other high-level networks^22–25^, and has been shown to reliably represent semantic information during language comprehension^11,26^. Here recordings were performed in participants undergoing planned intraoperative neurophysiology. Moreover, all participants were awake and therefore capable of performing language-based tasks, providing the unique opportunity to study the action potential dynamics of individual neurons during comprehension in humans.

Altogether, we recorded from 133 well-isolated single units (Fig. 1a, right, and Extended Data Fig. 1a,b) in 10 participants (18 sessions; 8 male and 2 female individuals, age range 33–79; Extended Data Table 1) using custom-adapted tungsten microelectrode arrays^27–29^ (microarray; Methods (‘Single-unit isolation’)). To further confirm the consistency and robustness of neuronal responses, an additional 154 units in 3 participants (3 sessions; 2 male individuals and 1 female individual; age range 66–70; Extended Data Table 1) were also recorded using silicon Neuropixels arrays^30,31^ (Methods (‘Single-unit isolation’) and Extended Data Fig. 1c,d) that allowed for higher-throughput recordings per participant (287 units across 13 participants in total; 133 units from the microarray recordings and 154 units from the Neuropixels recordings). All participants were right-hand-dominant native English speakers and were confirmed to have normal language function by preoperative testing.

During recordings, the participants listened to semantically diverse naturalistic sentences that were played to them in a random order. This amounted to an average of 459 ± 24 unique words or 1,052 ± 106 word tokens (± s.e.m) across 131 ± 13 sentences per participant (Methods (‘Linguistic materials’) and Extended Data Table 1). Additional controls included the presentations of unstructured word lists, nonwords and naturalistic story narratives (Extended Data Table 1). Action potential activities were aligned to each word or nonword using custom-made software at millisecond resolution and analysed off-line (Fig. 1b). All primary findings describe results for the tungsten microarray recordings unless stated otherwise for the Neuropixels recordings (Extended Data Fig. 1).

## Selectivity of neurons to specific word meanings

A long-standing observation^32^ that lies at the core of all distributional models of meaning^33^ is that words that share similar meanings tend to occur in similar contexts. Data-driven word embedding approaches that capture these relationships through vectoral representations^11,34–39^ have been found to estimate word meanings quite well and to accurately capture human behavioural semantic judgements^40^ and neural responses to meaning through brain-imaging studies^11,26,37,39,41^.

**Extended Data Fig. 2:**
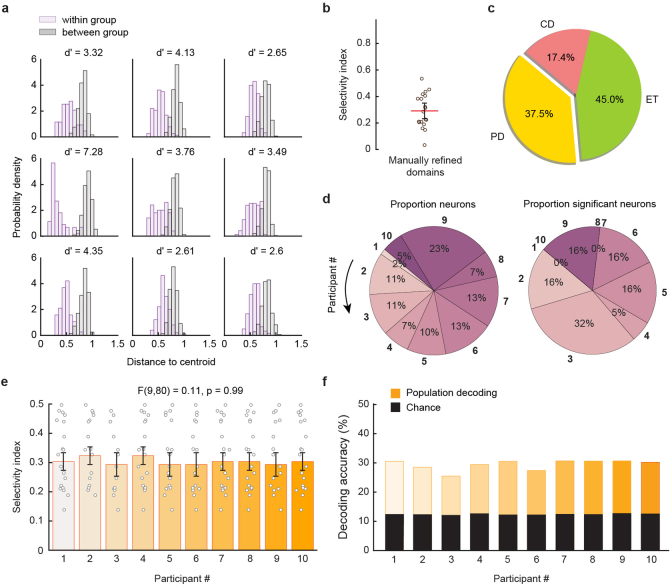
Cluster separability and consistency of neuronal responses across participants. **a**, The *d’* (*d*-prime) indices measuring separability between the distribution of the vectoral cosine distances among all words within a cluster (purple) and those among all words across clusters (gray). The d’ indices were all above 2.5 reflecting strong separability. **b**, Selectivity index of neurons (mean with 95% CL, *n* = 19) when semantic domains were refined by moving or removing words whose meanings did not intuitively fit with their respective labels (Extended Data Table 2). **c**, There was no significant difference (χ^2^ = 2.33, *p* = 0.31) in the proportions of neurons that displayed semantic selectivity based on the participants’ clinical conditions of essential tremor (ET), Parkinson’s disease (PD) or cervical dystonia (CD). **d**, *Left*, the proportional contribution *per* participant based on the total percentage of neurons contributed. *Right*, the proportional contribution of semantically selective cells *per* participant based on the fraction contributed. Participants without selective cells are not shown. **e**, A leave one out cross-validation participant-dropping procedure demonstrated that population results remained similar. Here, we sequentially removed individual participants (i.e., participants #1-10) and then repeated our selectivity analysis. Semantic selectivity across neurons was largely unaffected by removal of any of the participants (one-way ANOVA, *F*(9, 44) = 0.11, *p* = 0.99). Here, the mean selectivity indices (± s.e.m.) are separately presented after removing each participant. **f**, A cross-validation participant-dropping procedure was used to determine whether any of the participants disproportionately contributed to the population decoding. Average decoding results and comparison to the shuffled data are separately presented after removing each participant (permutation test, *p* < 0.01; #1-10).

To first examine whether and to what degree the activities of neurons within the population reflected the words’ meanings during speech processing, we used an embedding approach that replaced each unique word heard by the participants with pretrained 300-dimensional embedding vectors extracted from a large English corpus (Methods (‘Word embedding and clustering procedures’))^35,37,39,42^. Thus, for instance, the words ‘clouds’ and ‘rain’, which are closely related in meaning, would share a smaller vectoral cosine distance in this embedding space when compared to ‘rain’ and ‘dad’ (Fig. 1c, left). Next, to determine how the words optimally group into semantic domains, we used a spherical clustering and silhouette criterion analysis^34,37,43,44^ to reveal the following nine putative domains: actions (for example, ‘walked’, ‘ran’ and ‘threw’), states (for example, ‘happy’, ‘hurt’ and ‘sad’), objects (for example, ‘hat’, ‘broom’ and ‘lampshade’), food (for example, ‘salad’, ‘carrots’ and ‘cake’), animals (for example, ‘bunny’, ‘lizard’ and ‘horse’), nature (for example, ‘rain’, ‘clouds’ and ‘sun’), people and family (for example, ‘son’, ‘sister’ and ‘dad’), names (for example, ‘george’, ‘kevin’ and ‘hannah’) and spatiotemporal relationships (for example, ‘up’, ‘down’ and ‘behind’; Fig. 1c right and Extended Data Tables 2 and 3). Purity and *d*′ measures confirmed the quality and separability of these word clusters (Fig. 1d and Extended Data Fig. 2a).

**Extended Data Fig. 3:**
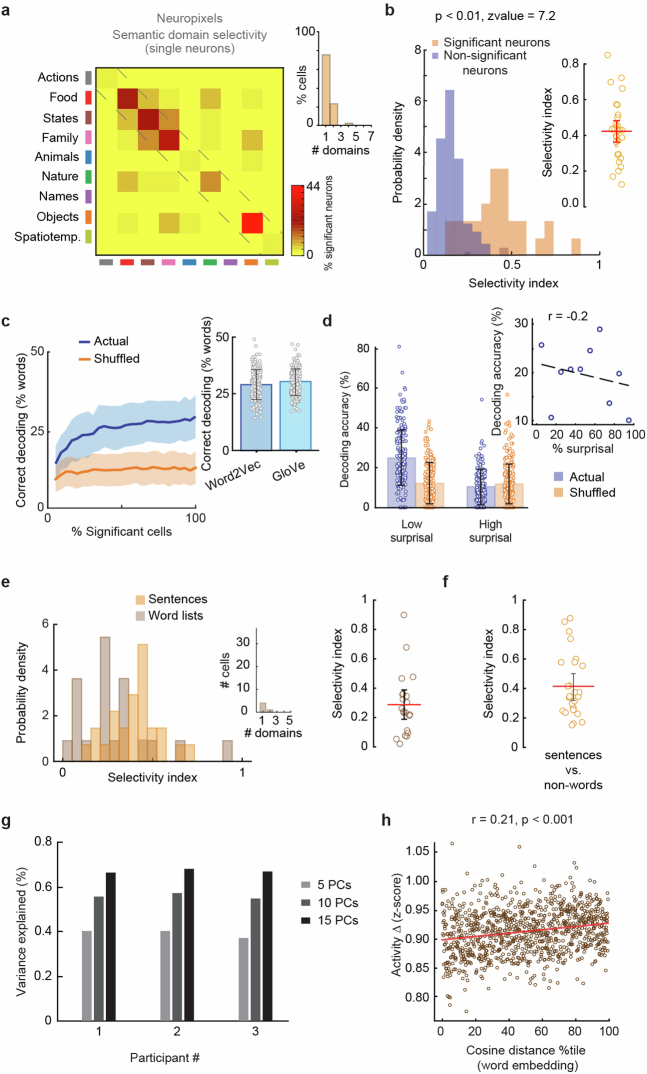
Confirming consistency of semantic representations by neurons using Neuropixels recordings. **a**, Coincidence matrix illustrating the distribution of cells obtained from Neuropixels recordings that displayed selective responses to one or more semantic domains (two-tailed rank-sum test, *p* < 0.05, FDR adjusted). *Inset*, proportions of cells that displayed selective responses to one or more semantic domains. **b**, The distributions of SIs are shown separately for semantically-selective (*n* = 29, orange) and non-selective (*n* = 125, grey) cells. The mean SI of cells that did not display semantic selectivity (*n* = 125) was 0.16 (one-sided rank-sum test, *z*-value = 7.2, *p* < 0.0001). *Inset*, selectivity index (SI) of each neuron (*n* = 29) when compared across different semantic domains. **c**, The cumulative decoding performance (± s.d.) of all semantically selective neurons during sentences (blue) versus chance (orange). *Inset*, decoding performances (± s.d.) across two independent embedding models (Word2Vec and GloVe). **d**, Decoding accuracies for words that displayed high vs. low surprisal based on the preceding sentence contexts in which they were heard. Actual and chance decoding performances are shown in blue and orange, respectively (mean ± s.d., one-sided rank-sum test *z*-value = 25, *p* < 0.001). The *inset* shows a regression analysis on the relation between decoding performance and surprisal. **e**, *Left*, SI distributions for neurons during word list and sentence presentations together with the number of neurons that responded selectivity to one or more semantic domains (*Inset*). *Right*, the SI for neurons (mean with 95% CL, *n* = 21; excluding zero firing rate neurons) during word-list presentation. The SI dropped from 0.39 (CI = 0.33-0.45) during the sentences to 0.29 (CI = 0.19-0.39) during word list presentation (signed-rank test, *z*(41) = 168, *p* = 0.035). **f**, The selectivity index of neurons for which nonword lists presentation was performed (*n* = 26 of 153 cells were selective) when comparing their activities during sentences vs. nonwords (mean SI = 0.34, CI = 0.28-0.40). Here, the selectivity of each neuron reflects the degree to which it differentiates any semantic (meaningful) compared to non-semantic (nonmeaningful) information. **g**, Contribution to the variance explained in PC space for word projections across participants using a participant-dropping procedure. **h**, Activities of neurons for word pairs based on their vectoral cosine distance within the 300-dimensional embedding space (*z*-scored activity change over percentile cosine similarity; Pearson’s correlation *r* = 0.21, *p* < 0.001).

We observed that many of the neurons responded selectively to specific word meanings. The selectivity or ‘tuning’ of neurons reflects the degree to which they respond to words denoting particular meanings (that is, words that belong to specific semantic domains). Thus, a selectivity index (SI) of 1.0 would indicate that a cell responded to words within only one semantic domain and no other, whereas an SI of 0 would indicate no selectivity (that is, similar responses to words across all domains; Methods (‘Evaluating the responses of neurons to semantic domains’)). Altogether, 14% (*n* = 19 of 133; microarray) of the neurons responded selectively to specific semantic domains indicating that their firing rates distinguished between words on the basis of their meanings (two-tailed rank-sum test comparing activity for each domain to that of all other domains; false discovery rate-corrected for the 9 domains, *P* < 0.05). Thus, for example, a neuron may respond selectively to ‘food’ items whereas another may respond selectively to ‘objects’ (Fig. 1e). The domain that elicited the largest change in activity for the largest number of cells was that of ‘actions’, and the domain that elicited changes for the fewest cells was ‘spatiotemporal relations’ (Fig. 1f). The mean SI across all selective neurons was 0.32 (*n* = 19; 95% confidence interval (CI) = 0.26–0.38; Fig. 1g, left) and progressively increased as the semantic domains became more specific in meaning (that is, when removing words that lay farther away from the domain centroid; analysis of variance, *F*(3,62) = 8.66, *P* < 0.001; Fig. 1h and Methods (‘Quantifying the specificity of neuronal response’)). Findings from the Neuropixels recordings were similar, with 19% (*n* = 29 of 154; Neuropixels) of the neurons exhibiting semantic selectivity (mean SI = 0.42, 95% CI = 0.36–0.48; Extended Data Fig. 3a,b), in aggregate, providing a total of 48 of 287 semantically selective neurons across the 13 participants. Many of the neurons across the participants and recording techniques therefore exhibited semantic selectivity during language comprehension.

Most of the neurons that exhibited semantic selectivity responded to only one semantic domain and no other. Of the neurons that demonstrated selectivity, 84% (*n* = 16; microarray) responded to one of the nine domains, with only 16% (*n* = 3) showing response selectivity to two domains (two-sided rank-sum test, *P* < 0.05; Fig. 1f, top right). The response selectivity of these neurons was also robust to analytic choice, demonstrating a similarly high degree of selectivity when randomly sub-selecting words (SI = 0.33, CI = 0.32–0.33, rank-sum test when compared to the original SI values, *z* value = 0.44, *P* = 0.66, Fig. 1g, top right, and Methods (‘Evaluating the responses of neurons to semantic domains’)) or when selecting words that intuitively fit within their respective domains (SI = 0.30; rank-sum test compared to the original SI values, *z* value = 0.60, *P* = 0.55; Extended Data Fig. 2b and Extended Data Table 2). Moreover, they exhibited a similarly high degree of selectivity when selecting nonadjacent content words (SI = 0.34, CI = 0.26–0.42; Methods), further confirming the consistency of neuronal response.

**Extended Data Fig. 4:**
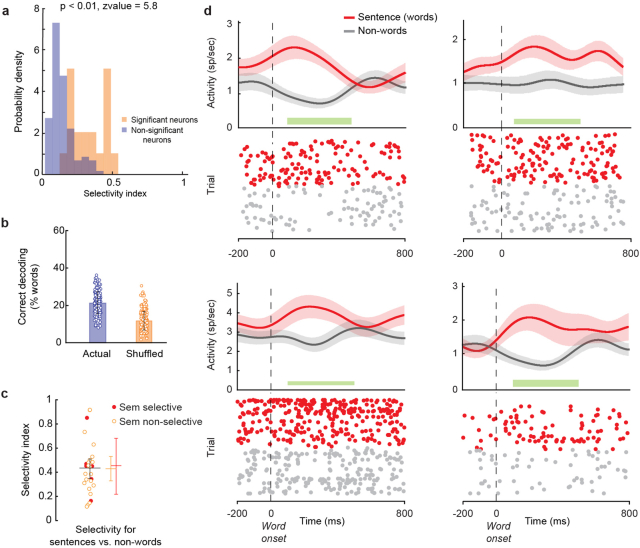
Selectivity of neurons to linguistically meaningful versus nonmeaningful information. **a**, The distributions of SIs are shown separately for cells that displayed significance for semantic information (*n* = 19, orange) and those that did not (*n* = 114, grey). The mean SI of cells that did not display semantic selectivity (*n* = 114) was 0.14 (one-sided rank-sum test, *z*-value = 5.8, *p* < 0.0001). **b**, Decoding performances (mean ± s.d.) for cells that were not significantly selective for any particular semantic domain but which had an SI greater than 0.2 (*n* = 11) compared to that of shuffled data (21 ± 6%; permutation test, *p* = 0.046). **c**, The selectivity index of neurons for which nonword lists presentation was performed (*n* = 27 of 48 cells for which this control was performed displayed a significant difference in activity using a two-sided *t*-test) when comparing their responses to nonwords (i.e., that carried no linguistic meaning) versus sentences (i.e., that carried linguistic meaning; mean SI = 0.43, CI = 0.35-0.51). The semantically selective cells (*n* = 6, red) displayed a similar word vs. nonword SI when compared to the non-semantically selective cells (*n* = 21, orange; two-sided *t*-test, *df* = 26, *p* = 1.0). **d**, Peristimulus histograms (mean ± s.e.m.) and rasters of representative neurons when the participants were given words heard within sentences (red) or sets of nonwords (gray). The horizontal green bars display the 400 ms window of analysis.

Finally, given these findings, we tested whether the neurons distinguished real words from nonwords (such as ‘blicket’ or ‘florp’, which sound like words but are meaningless), as might be expected of cells that represent meaning. Here we found that many neurons distinguished words from nonwords (27 of 48 neurons; microarray, in 7 participants for whom this control was carried out; two-tailed *t*-test, *P* < 0.05; Methods (‘Linguistic materials; Nonwords’)), meaning that they exhibited a consistent difference in their activities. Moreover, the ability to differentiate words from nonwords was not necessarily restricted to semantically selective neurons (Extended Data Fig. 3f, Neuropixels, and Extended Data Fig. 4, microarray), together revealing a broad mixture of response selectivity to word meanings within the cell population.

## Generalizable and robust meaning representations

**Fig. 2:**
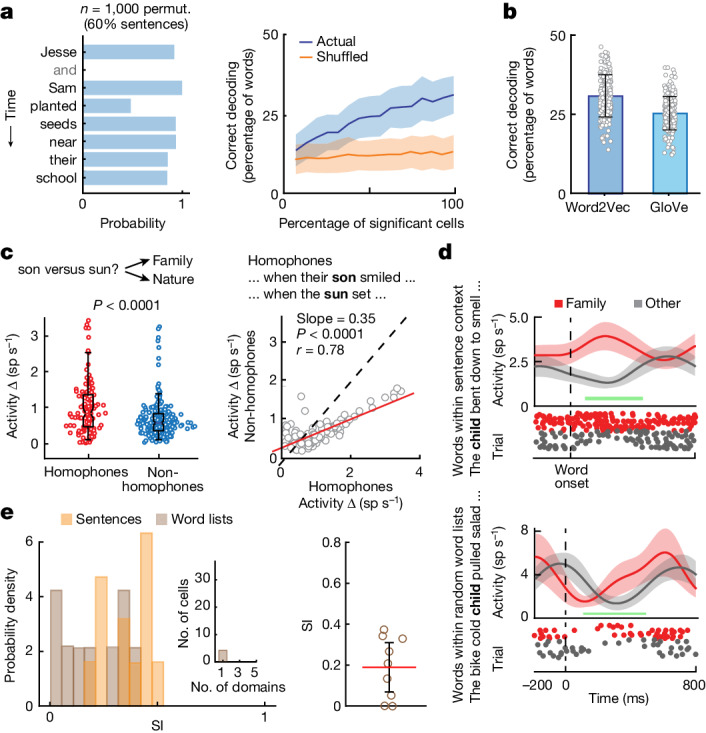
Decoding word meanings during language comprehension. **a**, Left: projected probabilities of correctly predicting the semantic domain to which individual words belonged over a representative sentence. Right: the cumulative decoding performance (±s.d.) of all semantically selective neurons during presentation of sentences (blue) versus chance (orange); see also Extended Data Fig. 4b. **b**, Decoding performances (±s.d.) across two independent embedding models (Word2Vec and GloVe). **c**, Left: the absolute difference in neuronal responses (*n* = 115) for homophone pairs that sounded the same but differed in meaning (red) compared to that of non-homophone pairs that sounded different but shared similar meanings (blue; two-sided permutation test). Right: scatter plot displaying each neuron’s absolute difference in activity for homophone versus non-homophone pairs (*P* < 0.0001, one-sided *t*-test comparing linear fit to identity line). **d**, Peri-stimulus spike histogram (mean ± s.e.m.) and raster from a representative neuron when hearing words within sentences (top) compared to words within random word lists (bottom). The horizontal green bars mark the window of analysis (100–500 ms from onset). **e**, Left: SI distributions for neurons during word-list and sentence presentations together with the number of neurons that responded selectivity to one or more semantic domains (inset). Right: the SI for neurons (mean with 95% confidence limit, *n* = 9; excluding zero firing rate neurons) during word-list presentation. These neurons did not exhibit changes in mean firing rates when comparing all sentences versus word lists independently of semantic domains (rank-sum test, *P* = 0.16).

**Extended Data Fig. 5:**
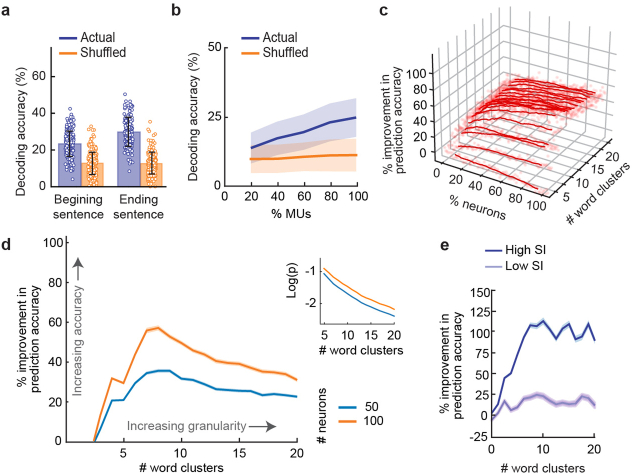
Generalizability and robustness of word meaning representations. **a**, Average decoding performances (± s.d., purple, *n* = 1000 iterations) were found to be slightly lower for words heard early (first 4 words) vs. late (last 4 words) within their respective sentences (23 ± 7% vs. 29 ± 8% decoding performance, respectively; One-sided rank sum test, *z*-value = 17, *p* < 0.001)^76,77^. The orange bars represent control accuracy with shuffling neuronal activities. **b**, Cumulative mean decoding performance (±s.d., purple) for multi-units (MUs) compared with chance (orange). The mean decoding accuracy for all MUs was 23 ± 6% s.d. (one-sided permutation test, *p* = 0.02) and reflect the unsorted activities of units obtained through recordings (**Methods**). **c**, Relationship between the number of neurons considered, the number of word clusters modeled, and prediction accuracy. Here, a lower number of clusters leads to more words per grouping and therefore domains that are not as specific in meaning (e.g., “*sun*”, “*rain*”, “*clouds*”, and “*sky*”,) whereas a higher number of clusters means fewer words and therefore domains that are more specific in meaning (e.g., “*rain*” and “*clouds*”). **d**, The percent improvement in decoding accuracy (mean ± s.e.m) corresponds to decoding performance minus chance probability using 60% of randomly selected sentences for modeling and 40% for decoding (*n* = 200 iterations). *Inset*, relation between log of odds probability (mean ± s.e.m) of predicting the correct semantic domains and number of clusters (i.e., not accounting for chance probability). **e**, The relation between the number of word clusters modeled and the percent improvement in decoding accuracy (mean ± s.e.m) when considering semantically selective (high SI) and non-selective (low SI) cells separately.

Meaning representations by the semantically selective neurons were robust. Training multi-class decoders on the combined response patterns of the semantically selective cells, we found that these cell ensembles could reliably predict the semantic domains of randomly selected subsets of words not used for training (31 ± 7% s.d.; chance: 11%, permutation test, *P* < 0.01; Fig. 2a and Methods (‘Model decoding performance and the robustness of neuronal response’)). Moreover, similar decoding performances were observed when using a different embedding model (GloVe^45^; 25 ± 5%; permutation test, *P* < 0.05; Fig. 2b) or when selecting different recorded time points within the sentences (that is, the first half versus the second half of the sentences; Extended Data Fig. 5a). Similar decoding performances were also observed when randomly subsampling neurons from across the population (Extended Data Fig. 5c–e), or when examining multi-unit activities for which no spike sorting was carried out (permutation test, *P* < 0.05; Methods (‘Multi-unit isolation’) and Extended Data Fig. 5b). In tandem, these analyses therefore suggested that the words’ meanings were robustly represented within the population’s response patterns.

**Extended Data Fig. 6:**
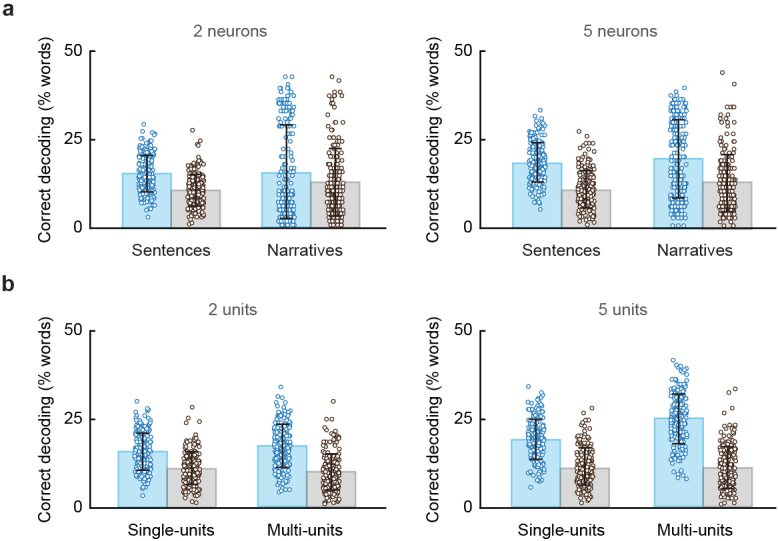
Semantic selectivity during naturalistic story narratives. **a**, Comparison of average decoding performances (± s.d., blue, *n* = 200 iterations) for sentences and naturalistic story narratives, matched based on the number of neurons (*left*: 2 neurons, *right*: 5 neurons). **b**, Comparison of average decoding performances (± s.d., blue, *n* = 200 iterations) for sentences, matched based on the number of single-units or multi-units (*left*: 2 units, *right*: 5 units). Chance decoding performances are given in gray.

We also examined whether the activities of the neurons could be generalized to an entirely new set of naturalistic narratives. Here, for three of the participants, we additionally introduced short story excerpts that were thematically and stylistically different from the sentences and that contained new words (Extended Data Table 1; 70 unique words of which 28 were shared with the sentences). We then used neuronal activity recorded during the presentation of sentences to decode semantic domains for words heard during these stories (Methods (‘Linguistic materials; Story narratives’)). We find that, even when using this limited subset of semantically selective neurons (*n* = 9; microarray), models that were originally trained on activity recorded during the presentation of sentences could predict the semantic domains of words heard during the narratives with significant accuracy (28 ± 5%; permutation test, *P* < 0.05; Extended Data Fig. 6).

Finally, to confirm the consistency of these semantic representations, we evaluated neuronal responses across the different participants and recording techniques. Here we found similar results across individuals (permutation test, *P* < 0.01) and clinical conditions (*χ*^2^ = 2.33, *P* = 0.31; Methods (‘Confirming the robustness of neuronal response across participants’) and Extended Data Fig. 2c–f), indicating that the results were not driven by any single participant or a small subset of participants. We also evaluated the consistency of semantic representations in the three participants who underwent Neuropixels recordings and found that the activities of semantically selective neurons in these participants could be used to reliably predict the semantic domains of words not used for model fitting (29 ± 7%; permutation test, *P* < 0.01; Extended Data Fig. 3c) and that they were comparable across embedding models (GloVe; 30 ± 6%). Collectively, decoding performance across the 13 participants (48 of 287 semantically selective neurons in total) was 36 ± 7% and significantly higher than expected from chance (permutation test, *P* < 0.01; Methods). These findings therefore together suggested that these meaning representations by semantically selective neurons were both generalizable and robust.

## Sentence context dependence of meaning encoding

An additional core property of language is our ability to interpret words on the basis of the sentence contexts in which they appear^46,47^. For example, hearing the sequences of words “He picked the rose…” versus “He finally rose…” allows us to correctly interpret the meaning of the ambiguous word ‘rose’ as a noun or a verb. It also allows us to differentiate homophones—words that sound the same but differ in meaning (such as ‘sun’ and ‘son*’*)—on the basis of their contexts.

Therefore, to first evaluate the degree to which the meaning representations by neurons are sentence context dependent, seven of the participants were presented with a word-list control that contains the same words as those heard in the sentences but were presented in random order (for example, “to pirate with in bike took is one”; Extended Data Table 1), thus largely removing the influence of context on lexical (word-level) processing. Here we find that, the SI of the neurons that exhibited semantic selectivity in the sentence condition dropped from a mean of 0.34 (*n* = 9 cells; microarray, CI = 0.25–0.43) to 0.19 (CI = 0.07–0.31) during the word-list presentation (signed-rank test, *z*(17) = 40, *P* = 0.02; Fig. 2d,e), in spite of similar mean population firing rate^48^ (two-sided rank-sum test, *z* value = 0.10, *P* = 0.16). The results were similar for the Neuropixels recordings, for the SI dropped from 0.39 (CI = 0.33–0.45) during the presentation of sentences to 0.29 (CI = 0.19–0.39) during word-list presentation (Extended Data Fig. 3e; signed-rank test, *z*(41) = 168, *P* = 0.035). These findings therefore suggested that the response selectivity of these neurons was strongly influenced by the word’s context and that these changes were independent of potential variations in attentional engagement, as evidenced by similar overall firing rates between the sentences and word lists^48^.

**Fig. 3:**
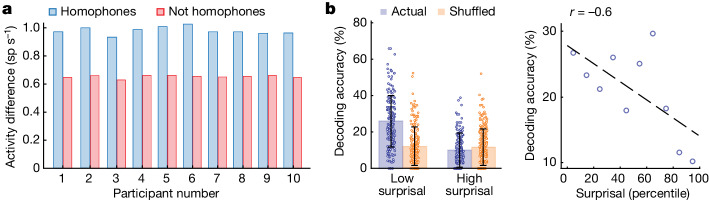
Sentence context dependence and word meaning predictions. **a**, Differences in neuronal activity comparing homophone (for example, ‘son’ and ‘sun’; blue) to non-homophone (for example, ‘son’ and ‘dad’; red) pairs across participants using a participant-dropping procedure (two-sided paired *t*-test, *P* < 0.001 for all participants). **b**, Left: decoding accuracies for words that showed high versus low surprisal based on the preceding sentence contexts in which they were heard. Words with lower surprisal were more predictable on the basis of their preceding word sequence. Actual and chance decoding performances are shown in blue and orange, respectively (mean ± s.d., one-sided rank-sum test, *z* value = 26, *P* < 0.001). Right: a regression analysis on the relation between decoding performance and surprisal.

Second, to test whether the neurons’ activity reflected the words’ meanings independently of their word-form similarity, we used homophone pairs that are phonetically identical but differ in meaning (for example, ‘sun’ versus ‘son’; Extended Data Table 1). Here we find that neurons across the population exhibited a larger difference in activity for words that sounded the same but had different meanings (that is, homophones) compared to words that sounded different but belonged to the same semantic domain (permutation test, *P* < 0.0001; *n* = 115 cells; microarray, for which data were available; Figs. 2c and 3a and Methods (‘Evaluating the context dependency of neuronal response using homophone pairs’)). These neurons therefore encoded the words’ meanings independently of their sound-level similarity.

Last, we quantified the degree to which the words’ meanings could be predicted from the sentences in which they appeared. Here we reasoned that words that were more likely to occur on the basis of their preceding word sequence and context should be easier to decode. Using a long short-term memory model to quantify each word’s surprisal based on its sentence context (Methods (‘Evaluating the context dependency of neuronal response using surprisal analysis’)), we find that decoding accuracies for words that were more predictable were significantly higher than for words that were less predictable (comparing top versus bottom deciles; 26 ± 14% versus 10 ± 9% respectively, rank-sum test, *z* value = 26, *P* < 0.0001; Fig. 3b). Similar findings were also obtained from the Neuropixels recordings (rank-sum test, *z* value = 25, *P* < 0.001; Extended Data Fig. 3d), indicating that information about the sentences was being tracked and that it influenced neuronal response. These findings therefore together suggested that the activities of these neurons were dynamic, reflecting processing of the words’ meanings based on their specific sentence contexts and independently of their phonetic form.

## Organization of semantic representations

**Fig. 4:**
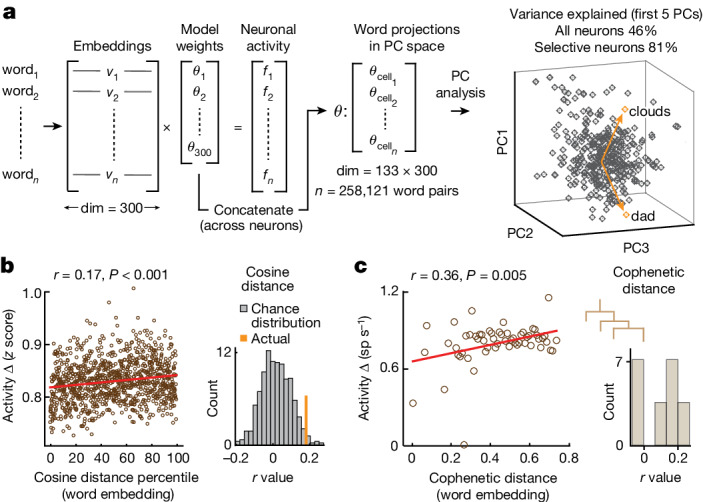
Hierarchical semantic relationship between word representations. **a**, Left: the activity of each neuron was regressed onto 300-dimensional word embedding vectors. A PC analysis was then used to dimensionally reduce this space from the concatenated set model parameters such that the cosine distance between each projection reflected the semantic relationship between words as represented by the neural population. Right: PC space with arrows highlighting two representative word projections. The explained variance and correlation between cosine distances for word projections derived from the word embedding space versus neural data (*n* = 258,121 possible word pairs) are shown in Extended Data Fig. 7a,b. **b**, Left: activities of neurons for word pairs based on their vectoral cosine distance within the 300-dimensional embedding space (*z*-scored activity change over percentile cosine similarity, red regression line; Pearson’s correlation, *r* = 0.17). Right: correlation between vectoral cosine distances in the word embedding space and difference in neuronal activity across possible word pairs (orange) versus chance distribution (grey, *n* = 1,000, *P* = 0.02; Extended Data Fig. 7c). **c**, Left: scatter plot showing the correlation between population-averaged neuronal activity and the cophenetic distances between words (*n* = 100 bins) derived from the word embedding space (red regression line; Pearson’s correlation, *r* = 0.36). Right: distribution of correlations between cophenetic distances and neuronal activity across the different participants (*n* = 10).

The above observations suggested that neurons within the population encoded information about the words’ meanings during comprehension. How they may represent the higher-order semantic relationships among words, however, remained unclear. Therefore, to further probe the organization of neuronal representations of meaning at the level of the cell population, we regressed the responses of the neurons (*n* = 133) onto the embedding vectors of all words in the study vocabulary (that is, a matrix of *n* words × 300 embedding dimensions), resulting in a set of model weights for the neurons (Fig. 4a, left, and Methods (‘Determining the relation between the word embedding space and neural response’)). These model weights were then concatenated (dimension = 133 × 300) to define a putative neuronal–semantic space. Each model weight can therefore be interpreted as the contribution of a particular dimension in the embedding space to the activity of a given neuron, such that the resulting transformation matrix reflects the semantic relationships among words as represented by the population^11,34,37^.

**Extended Data Fig. 7:**
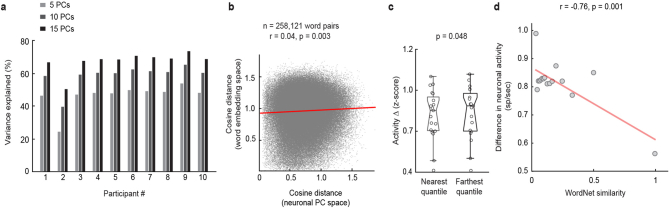
Population organization of semantic representations. **a**, Contribution to percent variance explained in PC space for word projections across participants using a participant-dropping procedure (first 5-15 PCs; two-sided *z*-test; *p* > 0.7). **b**, Correlation between the vectoral cosine distances between PC-reduced word-projections derived from the neural data and PC-reduced vectors derived from the 300-dimensional word embedding space (*n* = 258,121 possible word-pairs; note that not all pairs were used for all recordings *per* neuron since certain words were not heard by all participants). **c**, Difference in neuronal activities (*n* = 19 neurons, *p* = 0.048, two-sided paired *t*-test, *t*(18) = 2.12) for word pairs whose vectoral cosine distances were far versus near in the word embedding space. **d**, Relation between neuronal activity and word meaning similarity using a non-embedding based ‘synset’ approach (*n* = 100 bins, Pearson’s correlation *r* = −0.76, *p* = 0.001). Here, the degree of similarity ranges from 0 to 1.0, with a value of 1.0 indicating that the words are highly similar in meaning (e.g., “*canine*” and “*dog*”) and 0 indicating that their meanings are largely distinct.

Applying a principal component (PC) analysis to these weights, we find that the first five PCs accounted for 46% of the variance in neural population activity (Fig. 4a right and Extended Data Fig. 7a) and 81% of the variance for the semantically selective neurons (Extended Data Fig. 3g for the Neuropixels recordings). Moreover, when projecting words back into this PC space, we find that the vectoral distances between neuronal projections significantly correlated with the dimensionally reduced word distances in the original word embeddings (258,121 possible word pairings; *r* = 0.04, permutation test, *P* < 0.0001; Extended Data Fig. 7b). Significant correlations between word similarity and neuronal activity were also observed when using a non-embedding approach based on the ‘synset’ similarity metric (WordNet; *r* = −0.76, *P* = 0.001; Extended Data Fig. 7d) as well as when comparing the vectoral distances in the word embeddings to the raw firing activities of the neurons (*r* = 0.17; permutation test, one-sided, *P* = 0.02, Fig. 4b and Extended Data Fig. 7c for microarray recordings and *r* = 0.21; Pearson’s correlation, *P* < 0.001; Extended Data Fig. 3h for Neuropixels recordings). Our findings therefore suggested that these cell populations reliably captured the semantic relationships among words.

**Fig. 5:**
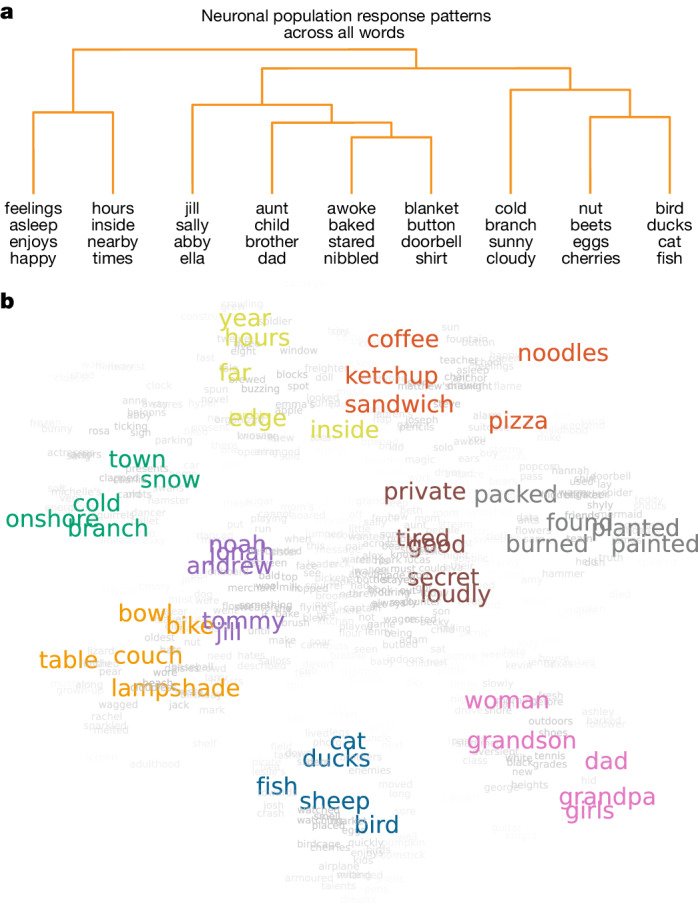
Organization of semantic representations within the cell population. **a**, An agglomerative hierarchical clustering procedure was carried out on all word projections in PC space obtained from the neuronal population data. The dendrogram shows representative word projections, with the branches truncated to allow for visualization. Words that were connected by fewer links in the hierarchy have a smaller cophenetic distance. **b**, A *t*-stochastic neighbour embedding procedure was used to visualize all word projections (in grey) by collapsing them onto a common two-dimensional manifold. For comparison, representative words are further colour-coded on the basis of their original semantic domain assignments in Fig. 1c.

Finally, to evaluate whether and to what degree neuronal activity reflected the hierarchical semantic relationship between words, we compared differences in firing activity for each word pair to the cophenetic distances between those words^49–51^ in the 300-dimension word embedding space (Methods (‘Estimating the hierarchical structure and relation between word projections’)). Here the cophenetic distance between a pair of words reflects the height of the dendrogram where the two branches that include these two words merge into a single branch. Using an agglomerative hierarchical clustering procedure, we find that the activities of the semantically selective neurons closely correlated with the cophenetic distances between words across the study vocabulary (*r* = 0.38, *P* = 0.004; Fig. 4c). Therefore, words that were connected by fewer links in the hierarchy and thus more likely to share semantic features (for example, ‘ducks’ and ‘eggs’)^50,51^ elicited smaller differences in activity than words that were connected by a larger number of links (for example, ‘eggs’ and ‘doorbell’; Fig. 5 and Methods (‘*t*-stochastic neighbour embedding procedure’)). These results therefore together suggested that these cell ensembles encoded richly detailed information about the hierarchical semantic relationship between words.

## Discussion

Neurons are the most basic computational units by which information is encoded in the brain. Yet, despite a growing understanding of the neural substrates of linguistic^4–12^ and semantic processing^11,37,41^, understanding how individual neurons represent semantic information during comprehension in humans has largely remained out of reach. Here, using single-neuronal recordings during natural speech processing, we discover cells in the prefrontal cortex of the language-dominant hemisphere that responded selectively to particular semantic domains and that exhibited preferential responses to specific word meanings. More notably, the combined activity patterns of these neurons could be used to accurately decode the semantic domain to which the words belonged even when tested across entirely different linguistic materials (that is, story narratives), suggesting a process that could allow semantic information to be reliably extracted during comprehension at the cellular scale. Lastly, to understand language, the meanings of words likely need to be robustly represented within the brain, entailing not only similar representations for words that share semantic features (for example, ‘mouse’ and ‘rat’) but also sufficiently distinct representations for words that differ in meaning (for example, ‘mouse’ and ‘carrot’). Here we find a putative cellular process that could support such robust word meaning representations during language comprehension.

Collectively, these findings imply that focal cortical areas such as the one from which we recorded here may be potentially able to represent complex meanings largely in their entirety. Although we sampled cells from a relatively restricted prefrontal region of the language-dominant hemisphere, these cell populations were capable of decoding meanings—at least at a relatively coarse level of semantic granularity—of a large set of diverse words and across independent sets of linguistic materials. The responses of these cell ensembles also harboured detailed information about the hierarchical relationship between words across thousands of word pairs, suggesting a cellular mechanism that could allow semantic information to be rapidly mapped onto the population’s response patterns, in real time during speech.

Another notable observation from these recordings is that the activities of the neurons were highly context dependent, reflecting the words’ meanings based on the specific sentences in which they were heard even when they were phonetically indistinguishable. Sentence context is essential to our ability to hone in on the precise meaning or aspects of meaning needed to infer complex ideas from linguistic utterances, and is proposed to play a key role in language comprehension^46,47,52^. Here we find that the neurons’ responses were highly dynamic, reflecting the meaning of the words within their respective contexts, even when the words were identical in form. Loss of sentence context or less predictive contexts, on the other hand, diminished the neurons’ ability to differentiate among semantic representations. Therefore, rather than simply responding to words as fixed stored memory representations, these neurons seemed to adaptively represent word meanings in a context-dependent manner during natural speech processing.

Taken together, these findings reveal a highly detailed representation of semantic information within prefrontal cortical populations, and a cellular process that could allow the meaning of words to be accurately decoded in real time during speech. As the present findings focus on auditory language processing, however, it is also interesting to speculate whether these semantic representations may be modality independent, generalizing to reading comprehension^53,54^, or even generalize to non-linguistic stimuli, such as pictures or videos or nonspeech sounds. Further, it remains to be discovered whether similar semantic representations would be observed across languages, including in bilingual speakers, and whether accessing word meanings in language comprehension and production would elicit similar responses (for example, whether the representations would be similar when participants understand the word ‘sun’ versus produce the word ‘sun’). It is also unknown whether similar semantic selectivity is present across other parts of the brain such as the temporal cortex, how finer-grained distinctions are represented, and how representations of specific words are composed into phrase- and sentence-level meanings.

Our study provides an initial framework for studying linguistic and semantic processing during comprehension at the level of individual neurons. It also highlights the potential benefit of using different recording techniques, linguistic materials and analytic techniques to evaluate the generalizability and robustness of neuronal responses. In particular, our study demonstrates that findings from the two recording approaches (tungsten microarray recordings and Neuropixels recordings) were highly concordant and suggests a platform from which to begin carrying out similar comparisons (especially in light of the increasing emphasis on robustness and replicability in the field). Collectively, our findings provide evidence of single neurons that encode word meanings during comprehension and a process that could support our ability to derive meaning from speech —opening the door for addressing a multitude of further questions about human-unique communicative abilities.

## Methods

### Study participants

All procedures and studies were carried out in accordance with the Massachusetts General Hospital Institutional Review Board and in strict adherence to Harvard Medical School guidelines. All participants included in the study were scheduled to undergo planned awake intraoperative neurophysiology and single-neuronal recordings for deep brain stimulation targeting. Consideration for surgery was made by a multidisciplinary team including neurologists, neurosurgeons and neuropsychologists^18,19,55–57^. The decision to carry out surgery was made independently of study candidacy or enrolment. Further, all microelectrode entry points and placements were based purely on planned clinical targeting and were made independently of any study consideration.

Once and only after a patient was consented and scheduled for surgery, their candidacy for participation in the study was reviewed with respect to the following inclusion criteria: 18 years of age or older, right-hand dominant, capacity to provide informed consent for study participation and demonstration of English fluency. To evaluate for language comprehension and the capacity to participate in the study, the participants were given randomly sampled sentences and were then asked questions about them (for example, “Eva placed a secret message in a bottle” followed by “What was placed in the bottle?”). Participants not able to answer all questions on testing were excluded from consideration. All participants gave informed consent to participate in the study and were free to withdraw at any point without consequence to clinical care. A total of 13 participants were enrolled (Extended Data Table 1). No participant blinding or randomization was used.

### Neuronal recordings

#### Acute intraoperative single-neuronal recordings

Microelectrode recording were performed in participants undergoing planned deep brain stimulator placement^19,58^. During standard intraoperative recordings before deep brain stimulator placement, microelectrode arrays are used to record neuronal activity. Before clinical recordings and deep brain stimulator placement, recordings were transiently made from the cortical ribbon at the planned clinical placement site. These recordings were largely centred along the superior posterior middle frontal gyrus within the dorsal prefrontal cortex of the language-dominant hemisphere. Here each participant’s computed tomography scan was co-registered to their magnetic resonance imaging scan, and a segmentation and normalization procedure was carried out to bring native brains into Montreal Neurological Institute space. Recording locations were then confirmed using SPM12 software and were visualized on a standard three-dimensional rendered brain (spm152). The Montreal Neurological Institute coordinates for recordings are provided in Extended Data Table 1, top.

We used two main approaches to perform single-neuronal recordings from the cortex^18,19^. Altogether, ten participants underwent recordings using tungsten microarrays (Neuroprobe, Alpha Omega Engineering) and three underwent recordings using linear silicon microelectrode arrays (Neuropixels, IMEC). For the tungsten microarray recordings, we incorporated a Food and Drug Administration-approved, biodegradable, fibrin sealant that was first placed temporarily between the cortical surface and the inner table of the skull (Tisseel, Baxter). Next, we incrementally advanced an array of up to five tungsten microelectrodes (500–1,500 kΩ; Alpha Omega Engineering) into the cortical ribbon at 10–100 µm increments to identify and isolate individual units. Once putative units were identified, the microelectrodes were held in position for a few minutes to confirm signal stability (we did not screen putative neurons for task responsiveness). Here neuronal signals were recorded using a Neuro Omega system (Alpha Omega Engineering) that sampled the neuronal data at 44 kHz. Neuronal signals were amplified, band-pass-filtered (300 Hz and 6 kHz) and stored off-line. Most individuals underwent two recording sessions. After neural recordings from the cortex were completed, subcortical neuronal recordings and deep brain stimulator placement proceeded as planned.

For the silicon microelectrode recordings, sterile Neuropixels probes^31^ (version 1.0-S, IMEC, ethylene oxide sterilized by BioSeal) were advanced into the cortical ribbon with a manipulator connected to a ROSA ONE Brain (Zimmer Biomet) robotic arm. The probes (width: 70 µm, length: 10 mm, thickness: 100 µm) consisted of 960 contact sites (384 preselected recording channels) that were laid out in a chequerboard pattern. A 3B2 IMEC headstage was connected via a multiplexed cable to a PXIe acquisition module card (IMEC), installed into a PXIe chassis (PXIe-1071 chassis, National Instruments). Neuropixels recordings were performed using OpenEphys (versions 0.5.3.1 and 0.6.0; https://open-ephys.org/) on a computer connected to the PXIe acquisition module recording the action potential band (band-pass-filtered from 0.3 to 10 kHz, sampled at 30 kHz) as well as the local field potential band (band-pass-filtered from 0.5 to 500 Hz, sampled at 2,500 Hz). Once putative units were identified, the Neuropixels probe was held in position briefly to confirm signal stability (we did not screen putative neurons for speech responsiveness). Additional description of this recording approach can be found in refs. ^20,30,31^. After completing single-neuronal recordings from the cortical ribbon, the Neuropixels probe was removed, and subcortical neuronal recordings and deep brain stimulator placement proceeded as planned.

#### Single-unit isolation

For the tungsten microarray recordings, putative units were identified and sorted off-line through a Plexon workstation. To allow for consistency across recording techniques (that is, with the Neuropixels recordings), a semi-automated valley-seeking approach was used to classify the action potential activities of putative neurons and only well-isolated single units were used. Here, the action potentials were sorted to allow for comparable isolation distances across recording techniques^59–63^ and unit selection with previous approaches^27–29,64,65^, and to limit the inclusion of multi-unit activity (MUA). Candidate clusters of putative neurons needed to clearly separate from channel noise, display a voltage waveform consistent with that of a cortical neuron, and have 99% or more of action potentials separated by an inter-spike interval of at least 1 ms (Extended Data Fig. 1b,d). Units with clear instability were removed and any extended periods (for example, greater than 20 sentences) of little to no spiking activity were excluded from the analysis. In total, 18 recording sessions were carried out, for an average of 5.4 units per session per multielectrode array (Extended Data Fig. 1a,b).

For the Neuropixels recordings, putative units were identified and sorted off-line using Kilosort and only well-isolated single units were used. We used Decentralized Registration of Electrophysiology Data (DREDge; https://github.com/evarol/DREDge) software and an interpolation approach (https://github.com/williamunoz/InterpolationAfterDREDge) to motion correct the signal using an automated protocol that tracked local field potential voltages using a decentralized correlation technique that realigned the recording channels in relation to brain movements^31,66^. Following this, we interpolated the continuous voltage data from the action potential band using the DREDge motion estimate to allow the activities of the recorded units to be stably tracked over time. Finally, putative neurons were identified from the motion-corrected interpolated signal using a semi-automated Kilosort spike sorting approach (version 1.0; https://github.com/cortex-lab/KiloSort) followed by Phy for cluster curation (version 2.0a1; https://github.com/cortex-lab/phy). Here, an *n*-trode approach was used to optimize the isolation of single units and limit the inclusion of MUA^67,68^. Units with clear instability were removed and any extended periods (for example, greater than 20 sentences) of little to no spiking activity were excluded from analysis. In total, 3 recording sessions were carried out, for an average of 51.3 units per session per multielectrode array (Extended Data Fig. 1c,d).

#### Multi-unit isolation

To provide comparison to the single-neuronal data, we also separately analysed MUA. These MUAs reflect the combined activities of multiple putative neurons recorded from the same electrodes as represented by their distinct waveforms^57,69,70^. These MUAs were obtained by separating all recorded spikes from their baseline noise. Unlike for the single units, the spikes were not separated on the basis of their waveform morphologies.

#### Audio presentation and recordings

The linguistic materials were given to the participants in audio format using a Python script utilizing the PyAudio library (version 0.2.11). Audio signals were sampled at 22 kHz using two microphones (Shure, PG48) that were integrated into the Alpha Omega rig for high-fidelity temporal alignment with neuronal data. Audio recordings were annotated in semi-automated fashion (Audacity; version 2.3). For the Neuropixels recordings, audio recordings were carried out at a 44 kHz sampling frequency (TASCAM DR-40× 4-channel 4-track portable audio recorder and USB interface with adjustable microphone). To further ensure granular time alignment for each word token with neuronal activity, the amplitude waveform of each session recording and the pre-recorded linguistic materials were cross-correlated to identify the time offset. Finally, for additional confirmation, the occurrence of each word token and its timing was validated manually. Together, these measures allowed for the millisecond-level alignment of neuronal activity with each word occurrence as they were heard by the participants during the tasks.

### Linguistic materials

#### Sentences

The participants were presented with eight-word-long sentences (for example, “The child bent down to smell the rose”; Extended Data Table 1) that provided a broad sample of semantically diverse words across a wide variety of thematic contents and contexts^4^. To confirm that the participants were paying attention, a brief prompt was used every 10–15 sentences asking them whether we could proceed with the next sentence (the participants generally responded within 1–2 seconds).

#### Homophone pairs

Homophone pairs were used to evaluate for meaning-specific changes in neural activity independently of phonetic content. All of the homophones came from sentence experiments in which homophones were available and in which the words within the homophone pairs came from different semantic domains. Homophones (for example, ‘sun’ and ‘son’; Extended Data Table 1), rather than homographs, were used as the word embeddings produce a unique vector for each unique token rather than for each token sense.

#### Word lists

A word-list control was used to evaluate the effect that sentence context had on neuronal response. These word lists (for example, “to pirate with in bike took is one”; Extended Data Table 1) contained the same words as those given during the presentation of sentences and were eight words long, but they were given in a random order, therefore removing any effect that linguistic context had on lexico-semantic processing.

#### Nonwords

A nonword control was used to evaluate the selectivity of neuronal responses to semantic (linguistically meaningful) versus non-semantic stimuli. Here the participants were given a set of nonwords such as ‘blicket’ or ‘florp’ (sets of eight) that sounded phonetically like words but held no meaning.

#### Story narratives

Excerpts from a story narrative were introduced at the end of recordings to evaluate for the consistency of neuronal response. Here, instead of the eight-word-long sentences, the participants were given a brief story about the life and history of Elvis Presley (for example, “At ten years old, I could not figure out what it was that this Elvis Presley guy had that the rest of us boys did not have”; Extended Data Table 1). This story was selected because it was naturalistic, contained new words, and was stylistically and thematically different from the preceding sentences.

### Word embedding and clustering procedures

#### Spectral clustering of semantic vectors

To study the selectivity of neurons to words within specific semantic domains, all unique words heard by the participants were clustered into groups using a word embedding approach^35,37,39,42^. Here we used 300-dimensional vectors extracted from a pretrained dataset generated using a skip-gram Word2Vec^11^ algorithm on a corpus of 100 billion words. Each unique word from the sentences was then paired with its corresponding vector in a case-insensitive fashion using the Python Gensim library (version 3.4.0; Fig. 1c, left). High unigram frequency words (log probability of greater than 2.5), such as ‘a’, ‘an’ or ‘and’, that held little linguistic meaning were removed.

Next, to group words heard by the participants into representative semantic domains, we used a spherical clustering algorithm (v.0.1.7, Python 3.6) that used the cosine distance between their representative vectors. We then carried out a *k*-means clustering procedure in this new space to obtain distinct word clusters. This approach therefore grouped words on the basis of their vectoral distance, reflecting the semantic relatedness between words^37,40^, which has been shown to work well for obtaining consistent word clusters^34,71^. Using pseudorandom initiation cluster seeding, the *k*-means procedure was repeated 100 times to generate a distribution of values for the optimal number of cluster. For each iteration, a silhouette criterion for cluster number between 5 and 20 was calculated. The cluster with the greatest average criterion value (as well as the most frequent value) was 9, which was taken as the optimal number of clusters for the linguistic materials used^34,37,43,44^.

#### Confirming the quality and separability of the semantic domains

Purity measures and *d*′ analysis were used to confirm the quality and separability of the semantic domains. To this end, we randomly sampled from 60% of the sentences across 100 iterations. We then grouped all words from these subsampled sentences into clusters using the same spherical clustering procedure described above. The new clusters were then matched to the original clusters by considering all possible matching arrangements and choosing the arrangement with greatest word overlap. Finally, the clustering quality was evaluated for ‘purity’, which is the percentage of the total number of words that were classified correctly^72^. This procedure is therefore a simple and transparent measure that varies between 0 (bad clustering) to 1 (perfect clustering; Fig. 1d, bottom). The accuracy of this assignment is determined by counting the total number of correctly assigned words and dividing by the total number of words in the new clusters:

$$\text{purity}\left(\Omega ,{\mathbb{C}}\right)=\frac{1}{n}\mathop{\sum }\limits_{i=1}^{k}{\max }_{j}\left|{\omega }_{i}\cap {c}_{j}\right|$$

in which *n* is the total number of words in the new clusters, *k* is the number of clusters (that is, 9), ${\omega }_{i}$ is a cluster from the set of new clusters $\Omega$, and ${c}_{j}$ is the original cluster (from the set of original clusters ${\mathbb{C}}$) that has the maximum count for cluster ${\omega }_{i}$. Finally, to confirm the separability of the clusters, we used a standard *d*′ analysis. The *d*′ metric estimates the difference between vectoral cosine distances for all words assigned to a particular cluster compared to those assigned to all other clusters (Extended Data Fig. 2a).

The resulting clusters were labelled here on the basis of the preponderance of words near the centroid of each cluster. Therefore, although not all words may seem to intuitively fit within each domain, the resulting semantic domains reflected the optimal vectoral clustering of words based on their semantic relatedness. To further allow for comparison, we also introduced refined semantic domains (Extended Data Table 2) in which the words provided within each cluster were additionally manually reassigned or removed by two independent study members on the basis of their subjective semantic relatedness. Thus, for example, under the semantic domain labelled ‘animals’, any word that did not refer to an animal was removed.

### Neuronal analysis

#### Evaluating the responses of neurons to semantic domains

To evaluate the selectivity of neurons to words within the different semantic domains, we calculated their firing rates aligned to each word onset. To determine significance, we compared the activity of each neuron for words that belonged to a particular semantic domain (for example, ‘food’) to that for words from all other semantic domains (for example, all domains except for ‘food’). Using a two-sided rank-sum test, we then evaluated whether activity for words in that semantic domain was significantly different from activity in all semantic domains, with the *P* value being false discovery rate-adjusted using a Benjamini–Hochberg method to account for repeated comparisons across all of the nine domains. Thus, for example, when stating that a neuron exhibited significant selectivity to the domain of ‘food’, this meant that it exhibited a significant difference in its activity for words within that domain when compared to all other words (that is, it responded selectively to words that described food items).

Next we determined the SI of each neuron, which quantified the degree to which it responded to words within specific semantic domains compared to the others. Here SI was defined by the cell’s ability to differentiate words within a particular semantic domain (for example, ‘food’) compared to all others and reflected the degree of modulation. The SI for each neuron was calculated as

$${\rm{SI}}=\frac{\left|{{\rm{FR}}}_{{\rm{domain}}}-{{\rm{FR}}}_{{\rm{other}}}\right|}{\left|{{\rm{FR}}}_{{\rm{domain}}}+{{\rm{FR}}}_{{\rm{other}}}\right|}$$

in which ${{\rm{FR}}}_{{\rm{domain}}}$ is the neuron’s average firing rate in response to words within the considered domain and ${{\rm{FR}}}_{{\rm{other}}}$ is the average firing rate in response to words outside the considered domain. The SI therefore reflects the magnitude of effect based on the absolute difference in activity for each neuron’s preferred semantic domain compared to others. Therefore, the output of the function is bounded by 0 and 1. An SI of 0 would mean that there is no difference in activity across any of the semantic domains (that is, the neuron exhibits no selectivity) whereas an SI of 1.0 would indicate that a neuron changed its action potential activity only when hearing words within one of the semantic domains.

A bootstrap analysis was used to further confirm reliability of each neuron’s SI across linguistic materials in two parts. For the first approach, the words were randomly split into 60:40% subsets (repeated 100 times) and the SI of semantically selective neurons was compared in both subsets of words. For the second, instead of using the mean SI, we calculated the proportion of times that a neuron exhibited selectivity for another category other than their preferred domain when randomly selecting words from 60% of the sentences.

#### Confirming the consistency of neuronal response across analysis windows

The consistency of neuronal response across analysis windows was confirmed in two parts. The average time interval between the beginning of one word and the next was 341 ± 5 ms. For all primary analysis, neuronal responses were analysed in 400-ms windows, aligned to each word, with a 100-ms time-lag to further account for the evoked response delay of prefrontal neurons. To further confirm the consistency of semantic selectivity, we first examined neuronal responses using 350-ms and 450-ms time windows. Combining recordings across all 13 participants, a similar proportion of cells exhibiting selectivity was observed when varying the window size by ±50 ms (17% and 15%, *χ*^2^(1, 861) = 0.43, *P* = 0.81) suggesting that the precise window of analysis did not markedly affect these results. Second, we confirmed that possible overlap between words did not affect neuronal selectivity by repeating our analyses but now evaluated only non-neighbouring content words within each sentence. Thus, for example, for the sentence “The child bent down to smell the rose”, we would evaluate only non-neighbouring words (for example, child, down and so on) per sentence. Using this approach, we find that the SI for non-overlapping windows (that is, every other word) was not significantly different from the original SIs (0.41 ± 0.03 versus 0.38 ± 0.02, *t* = 0.73, *P* = 0.47); together confirming that potential overlap between words did not affect the observed selectivity.

#### Model decoding performance and the robustness of neuronal response

To evaluate the degree to which semantic domains could be predicted from neuronal activity on a per-word level, we randomly sampled words from 60% of the sentences and then used the remaining 40% for validation across 1,000 iterations. Only candidate neurons that exhibited significant semantic selectivity and for which sufficient words and sentences were recorded were used for decoding purposes (43 of 48 total selective neurons). For these, we concatenated all of the candidate neurons from all participants together with their firing rates as independent variables, and predicted the semantic domains of words (dependent variable). Support vector classifiers (SVCs) were then used to predict the semantic domains to which the validation words belonged. These SVCs were constructed to find the optimal hyperplanes that best separated the data by performing

$$\mathop{min}\limits_{w,b,\zeta }\left(\frac{1}{2}{w}^{{\rm{T}}}{\rm{w}}+{\rm{C}}\mathop{\sum }\limits_{{\rm{i}}=1}^{{\rm{n}}}{\zeta }_{{\rm{i}}}\right)$$

subject to

$${y}_{i}({w}^{{\rm{T}}}\varphi ({x}_{i})+b)\,\ge 1-{\zeta }_{i}$$

in which $y\in {\left\{1,-1\right\}}^{n}$, corresponding to the classification of individual words, $x$ is the neural activity, and ${{\rm{\zeta }}}_{i}=\max \left(0,\,1-{y}_{i}\left(w{x}_{i}-b\right)\right)$. The regularization parameter *C* was set to 1. We used a linear kernel and ‘balanced’ class weight to account for the inhomogeneous distribution of words across the different domains. Finally, after the SVCs were modelled on the bootstrapped training data, decoding accuracy for the models was determined by using words randomly sampled and bootstrapped from the validation data. We further generated a null distribution by calculating the accuracy of the classifier after randomly shuffling the cluster labels on 1,000 different permutations of the dataset. These models therefore together determine the most likely semantic domain from the combined activity patterns of all selective neurons. An empirical *P* value was then calculated as the percentage of permutations for which the decoding accuracy from the shuffled data was greater than the average score obtained using the original data. The statistical significance was determined at *P* value < 0.05.

#### Quantifying the specificity of neuronal response

To quantify the specificity of neuronal response, we carried out two procedures. First, we reduce the number of words from each domain from 100% to 25% on the basis of their vectoral cosine distance from each of their respective domains’ centroid. Thus, for each domain, words that were closest to its centroid, and therefore most similar in meaning, were kept whereas words farther away were removed. The SIs of the neurons were then recalculated as before (Fig. 1h). Second, we repeated the decoding procedure but now varied the number of semantic domains from 2 to 20. Thus, a higher number of domains would mean fewer words per domain (that is, increased specificity of meaning relatedness) whereas a smaller number of domains would mean more words per domain. These decoders used 60% of words for model training and 40% for validation (200 iterations). Next, to evaluate the degree to which neuron and domain number led to improvement in decoding performance, models were trained for all combinations of domain numbers (2 to 20) and neuron numbers (1 to 133) using a nested loop. For control comparison, we repeated the decoding analysis but randomly shuffled the relation between neuronal response and each word as above. The percentage improvement in prediction accuracy (PA) for a given domain number (*d*) and neuronal size (*n*) was calculated as

$${\rm{improvement}}\left(d,\,n\right)=100\times \frac{\left[{{\rm{PA}}}_{{\rm{actual}}}\left(d,\,n\right)-{{\rm{PA}}}_{{\rm{shuffle}}}\left(d,\,n\right)\right]}{{{\rm{PA}}}_{{\rm{actual}}}\left(d,\,n\right)}$$

#### Evaluating the context dependency of neuronal response using homophone pairs

We compared the responses of neurons to homophone pairs to evaluate the context dependency of neuronal response and to further confirm the specificity of meaning representations. For example, if the neurons simply responded to differences in phonetic input rather than meaning, then we should expect to see smaller differences in firing rate between homophone pairs that sounded the same but differed in meaning (for example, ‘sun’ and ‘son’) compared to non-homophone pairs that sounded different but shared similar meaning (for example, ‘son’ and ‘sister’). Here, only homophones that belonged to different semantic domains were included for analysis. A permutation test was used to compare the distributions of the absolute difference in firing rates between homophone pairs (sample x) and non-homophone pairs (sample y) across semantically selective cells (*P* < 0.01). To carry out the permutation test, we first calculated the mean difference between the two distributions (sample x and y) as the test statistic. Then, we pooled all of the measurements from both samples into a single dataset and randomly divided it into two new samples x′ and y′ of the same size as the original samples. We repeated this process 10,000 times, each time computing the difference in the mean of x′ and y′ to create a distribution of possible differences under the null hypothesis. Finally, we computed the two-sided *P* value as the proportion of permutations for which the absolute difference was greater than or equal to the absolute value of the test statistic. A one-tailed *t*-test was used to further evaluate for differences in the distribution of firing rates for homophones versus non-homophone pairs (*P* < 0.001). To allow for comparison, 2 of the 133 neurons did not have homophone trials and were therefore excluded from analysis. An additional 16 neurons were also excluded for lack of response and/or for lying outside (>2.5 times) the interquartile range.

#### Evaluating the context dependency of neuronal response using surprisal analysis

Information theoretic metrics such as ‘surprisal’ define the degree to which a word can be predicted on the basis of its antecedent sentence context. To examine how the preceding context of each word modulated neuronal response on a per-word level, we quantified the surprisal of each word as follows:

$${\rm{surprisal}}\left({w}_{i}\right)=-\log P({w}_{i}{\rm{| }}{w}_{1}\ldots {w}_{i-1})$$

in which *P* represents the probability of the current word (*w*) at position *i* within a sentence. Here, a pretrained long short-term memory recurrent neural network was used to estimate *P*(*w*_*i*_ | *w*_1_*…w*_*i*−1_)^73^. Words that are more predictable on the basis of their preceding context would therefore have a low surprisal whereas words that are poorly predictable would have a high surprisal.

Next we examined how surprisal affected the ability of the neurons to accurately predict the correct semantic domains on a per-word level. To this end, we used SVC models similar to that described above, but now divided decoding performances between words that exhibited high versus low surprisal. Therefore, if the meaning representations of words were indeed modulated by sentence context, words that are more predictable on the basis of their preceding context should exhibit a higher decoding performance (that is, we should be able to predict their correct meaning more accurately from neuronal response).

#### Determining the relation between the word embedding space and neural response

To evaluate the organization of semantic representations within the neural population, we regressed the activity of each neuron onto the 300-dimensional embedded vectors. The normalized firing rate of each neuron was modelled as a linear combination of word embedding elements such that

$${F}_{i,w}={v}_{w}{\theta }_{i}+{\varepsilon }_{i}$$

in which ${F}_{i,w}$ is the firing rate of the *i*th neuron aligned to the onset of each word *w*, ${\theta }_{i}$ is a column vector of optimized linear regression coefficients, ${v}_{w}$ is the 300-dimensional word embedding row vector associated with word *w*, and ${\varepsilon }_{i}$ is the residual for the model. On a per-neuron basis, ${\theta }_{i}$ was estimated using regularized linear regression that was trained using least-squares error calculation with a ridge penalization parameter *λ* = 0.0001. The model values, ${\theta }_{i}$, of each neuron (dimension = 1 × 300) were then concatenated (dimension = 133 × 300) to define a putative neuronal–semantic space **θ**. Together, these can therefore be interpreted as the contribution of a particular dimension in the embedding space to the activity of a given neuron, such that the resulting transformation matrix reflects the semantic space represented by the neuronal population.

Finally, a PC analysis was used to dimensionally reduce **θ** along the neuronal dimension. This resulted in an intermediately reduced space (**θ**_pca_) consisting of five PCs, each with dimension = 300, together accounting for approximately 46% of the explained variance (81% for the semantically selective neurons). As this procedure preserved the dimension with respect to the embedding length, the relative positions of words within this space could therefore be determined by projecting word embeddings along each of the PCs. Last, to quantify the degree to which the relation between word projections derived from this PC space (neuronal data) correlated with those derived from the word embedding space (English word corpus), we calculated their correlation across all word pairs. From a possible 258,121 word pairs (the availability of specific word pairs differed across participants), we compared the cosine distances between neuronal and word embedding projections.

#### Estimating the hierarchical structure and relation between word projections

As word projections in our PC space were vectoral representations, we could also calculate their hierarchical relations. Here we carried out an agglomerative single-linkage (that is, nearest neighbour) hierarchical clustering procedure to construct a dendrogram that represented the semantic relationships between all word projections in our PC space. We also investigated the correlation between the cophenetic distance in the word embedding space and difference in neuronal activity across all word pairs. The cophenetic distance between a word pair is a measure of inter-cluster dissimilarity and is defined as the distance between the largest two clusters that contain the two words individually when they are merged into a single cluster that contains both^49–51^. Intuitively, the cophenetic distance between a word pair reflects the height of the dendrogram where the two branches that include these two words merge into a single branch. Therefore, to further evaluate whether and to what degree neuronal activity reflected the hierarchical semantic relationship between words, as observed in English, we also examined the cophenetic distances in the 300-dimension word embedding space. For each word pair, we calculated the difference in neuronal activity (that is, the absolute difference between average normalized firing rates for these words across the population) and then assessed how these differences correlated with the cophenetic distances between words derived from the word embedding space. These analyses were performed on the population of semantically selective neurons (*n* = 19). For further individual participant comparisons, the cophenetic distances were binned more finely and outliers were excluded to allow for comparison across participants.

#### *t*-stochastic neighbour embedding procedure

To visualize the organization of word projections obtained from the PC analysis at the level of the population (*n* = 133), we carried out a *t-*distributed stochastic neighbour embedding procedure that transformed each word projection into a new two-dimensional embedding space **θ**_tsne_ (ref. ^74^). This transformation utilized cosine distances between word projections as derived from the neural data.

#### Non-embedding approach for quantifying the semantic relationship between words

To further validate our results using a non-embedding approach, we used WordNet similarity metrics^75^. Unlike embedding approaches, which are based on the modelling of vast language corpora, WordNet is a database of semantic relationships whereby words are organized into ‘synsets’ on the basis of similarities in their meaning (for example, ‘canine’ is a hypernym of ‘dog’ but ‘dog’ is also a coordinate term of ‘wolf’ and so on). Therefore, although synsets do not provide vectoral representations that can be used to evaluate neuronal response to specific semantic domains, they do provide a quantifiable measure of word similarity^75^ that can be regressed onto neuronal activity.

#### Confirming the robustness of neuronal response across participants

Finally, to ensure that our results were not driven by any particular participant(s), we carried out a leave-one-out cross-validation participant-dropping procedure. Here we repeated several of the analyses described above but now sequentially removed individual participants (that is, participants 1–10) across 1,000 iterations. Therefore, if any particular participant or group of participants disproportionally contributed to the results, their removal would significantly affect them (one-way analysis of variance, *P* < 0.05). A *χ*^2^ test (*P* < 0.05) was used to further evaluate for differences in the distribution of neurons across participants.

### Reporting summary

Further information on research design is available in the Nature Portfolio Reporting Summary linked to this article.

## Online content

Any methods, additional references, Nature Portfolio reporting summaries, source data, extended data, supplementary information, acknowledgements, peer review information; details of author contributions and competing interests; and statements of data and code availability are available at 10.1038/s41586-024-07643-2.

### Supplementary information

Reporting Summary

## Figures and Tables

**Extended Data Table 1:**
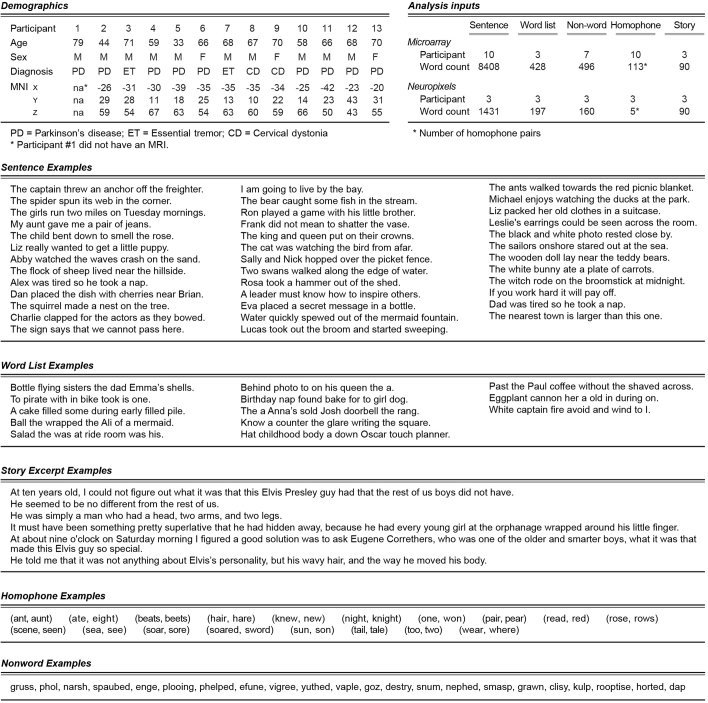
Demographic data of study participants and task materials

For demographics, the participants were recruited for participation in the study independently of underlying neuropathology, age, or sex. The corresponding recording coordinates for each participant are provided in MNI space (spm12). Participants 1-10 underwent tungsten microarray recordings and participants 11-13 underwent Neuropixels recordings. For the linguistic materials that were provided, the participants were given 8-word sentences that were made to be semantically diverse and were randomly interleaved over the course of recordings. Homophone pairs (i.e., word pairs with different spelling and meaning but with the same pronunciation) were interspersed throughout the sentences. The word lists contained the same words as those given during sentences and were 8 words long but, instead, were given in random order. The story excerpts were distinct in content and theme from that of the sentences and varied in length. The nonwords (i.e., elements that sound like words but hold no meaning) were given in sets of 8. The analysis inputs table provides the exact number of words used in each analysis across participants for the tungsten microarray (above) and Neuropixels (below) recordings.

**Extended Data Table 2:**
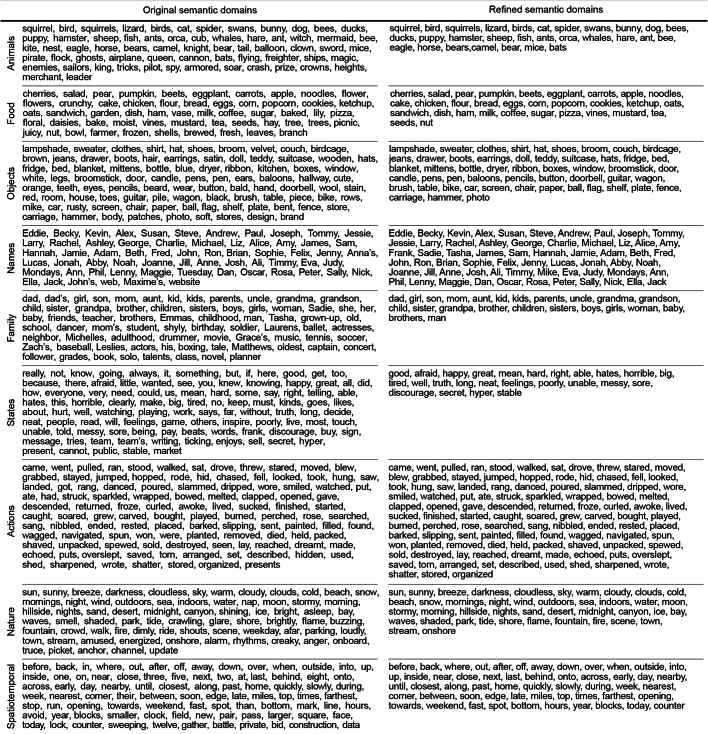
Semantic domains and word labels

*Left*, grouping of words into semantic domains based on their 300-dimensional embeddings was performed through a spherical clustering and silhouette criterion analysis (Fig. 1c,d). Here, the labels were selected based on the preponderance of words within each grouping and were ordered based on the proximity of each word to their respective cluster centroid. Words obtained from the linguistic materials given to the participants optimally grouped into nine putative semantic domains. *Right*, to further confirm the selectivity of neuronal response (Extended Data Fig. 2b), words that were not intuitively related in meaning *prima facie* were either moved or removed to produce the following refined semantic domains.

**Extended Data Table 3:**
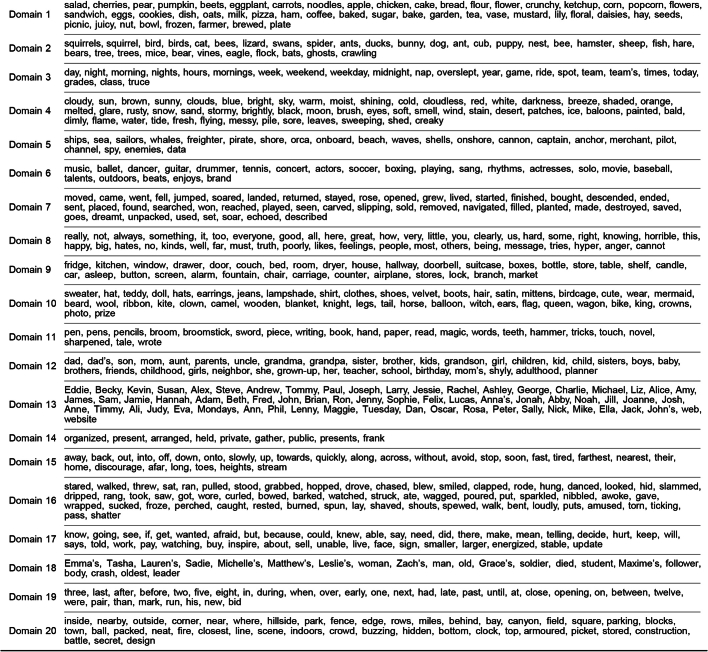
Grouping words into smaller word clusters

To further evaluate the selectivity of neuronal response, words were grouped into 20 rather than 9 clusters. As in Extended Data Table 2, the words were grouped using a spherical clustering and silhouette criterion analysis. Here, however, the words within each domain were closer in meaning since there were fewer words per cluster. Extended Data Fig. 4c–e provides further illustration of the relation between the number of clusters and neuronal decoding performance.

## Data Availability

All primary data supporting the findings of this study are available online at https://figshare.com/s/94962977e0cc8b405ef3. Details of the participants’ demographics and task conditions are provided in Extended Data Table 1.

## Code availability

All primary Python codes supporting the findings of this study are available online at https://figshare.com/s/94962977e0cc8b405ef3. Software packages used in this study are listed in the Nature Portfolio Reporting Summary along with their versions.
