# Supplementary material for: Semantic encoding during language comprehension at single-cell resolution
Source: Nature. 2024 Jul 3;631(8021):610–6. doi: 10.1038/s41586-024-07643-2 (PMC11254762; doi:10.1038/s41586-024-07643-2)
Supplement: Supplementary file 1 — Reporting Summary [file 41586_2024_7643_MOESM1_ESM.pdf]

nature research

Corresponding author(s): Ziv Williams

Last updated by author(s): Apr 5, 2024

Reporting Summary

Nature Research wishes to improve the reproducibility of the work that we publish. This form provides structure for consistency and transparency in reporting. For further information on Nature Research policies, see our Editorial Policies and the Editorial Policy Checklist.

Statistics

For all statistical analyses, confirm that the following items are present in the figure legend, table legend, main text, or Methods section.

n/a	Confirmed	
☐	☒	The exact sample size (*n*) for each experimental group/condition, given as a discrete number and unit of measurement
☐	☒	A statement on whether measurements were taken from distinct samples or whether the same sample was measured repeatedly
☐	☒	The statistical test(s) used AND whether they are one- or two-sided<br>*Only common tests should be described solely by name; describe more complex techniques in the Methods section.*
☐	☒	A description of all covariates tested
☐	☒	A description of any assumptions or corrections, such as tests of normality and adjustment for multiple comparisons
☐	☒	A full description of the statistical parameters including central tendency (e.g. means) or other basic estimates (e.g. regression coefficient) AND variation (e.g. standard deviation) or associated estimates of uncertainty (e.g. confidence intervals)
☐	☒	For null hypothesis testing, the test statistic (e.g. F, t, r) with confidence intervals, effect sizes, degrees of freedom and P value noted<br>*Give P values as exact values whenever suitable.*
☒	☐	For Bayesian analysis, information on the choice of priors and Markov chain Monte Carlo settings
☐	☒	For hierarchical and complex designs, identification of the appropriate level for tests and full reporting of outcomes
☐	☒	Estimates of effect sizes (e.g. Cohen's *d*, Pearson's *r*), indicating how they were calculated

Our web collection on statistics for biologists contains articles on many of the points above.

Software and code

Policy information about availability of computer code

Data collection	AlphaOmega and OpenEphys
Data analysis	Custom-written PYTHON scripts

For manuscripts utilizing custom algorithms or software that are central to the research but not yet described in published literature, software must be made available to editors and reviewers. We strongly encourage code deposition in a community repository (e.g. GitHub). See the Nature Research guidelines for submitting code & software for further information.

Data

Policy information about availability of data

All manuscripts must include a data availability statement. This statement should provide the following information, where applicable:
- Accession codes, unique identifiers, or web links for publicly available datasets
- A list of figures that have associated raw data
- A description of any restrictions on data availability

The data and primary codes that support the findings of this study are available from the corresponding author and will be deposited into Figshare once the study is published

Field-specific reporting

Please select the one below that is the best fit for your research. If you are not sure, read the appropriate sections before making your selection.

☒ Life sciences ☐ Behavioural & social sciences ☐ Ecological, evolutionary & environmental sciences

For a reference copy of the document with all sections, see nature.com/documents/nr-reporting-summary-flat.pdf

Life sciences study design

All studies must disclose on these points even when the disclosure is negative.

Sample size	A total of 13 participants underwent single-neuronal recordings. Our main neuronal analysis is based on data from 287 neurons. The sample size is large enough for the statistical analyses we have performed in our study consistent with previously published data.
Data exclusions	No subjects were excluded from analysis. For neuronal analysis, prospective units that did not demonstrate sufficient waveform stability over the course of the experiment were excluded from the analysis based on standard criteria for off-line single unit sorting. For Neuropixels recordings, units that displayed overlap with neighboring across channels were excluded as well.
Replication	Primary results of the study were replicated across participants, linguistic materials and recording techniques. For recording techniques, we used tungsten microarrays and Neuropixels probes in different participants performing the same language tasks. Additional analyses were also used to confirm generalizability of the findings across analytic techniques and through data sub-sampling. Other analyses were performed at the population level whereby semantic information was decoded from neuronal activity not used for model training. Finally, variability in results is shown through the plotting of individual data points or data ranges in the figures as applicable.
Randomization	There was no randomization procedure for subject selection/enrollment since all participants performed the same task.
Blinding	Blinding of analysis was not relevant since all subjects underwent similar task design. Blinding to clinical condition was performed.

Reporting for specific materials, systems and methods

We require information from authors about some types of materials, experimental systems and methods used in many studies. Here, indicate whether each material, system or method listed is relevant to your study. If you are not sure if a list item applies to your research, read the appropriate section before selecting a response.

Materials & experimental systems

n/a	Involved in the study	
☒	☐	Antibodies
☒	☐	Eukaryotic cell lines
☒	☐	Palaeontology and archaeology
☒	☐	Animals and other organisms
☐	☒	Human research participants
☒	☐	Clinical data
☒	☐	Dual use research of concern

Methods

n/a	Involved in the study	
☒	☐	ChIP-seq
☒	☐	Flow cytometry
☒	☐	MRI-based neuroimaging

Human research participants

Policy information about studies involving human research participants

Population characteristics	The participants were recruited for the study independently of underlying neuropathology, age or sex. The participants were drawn from the same population undergoing planned intraoperative neurophysiology for deep brain stimulator placement.
Recruitment	All procedures and studies were performed in accordance with the Massachusetts General Hospital Institutional Review Board (IRB) and were held in strict adherence to Harvard Medical School guidelines. All participants included in the study were scheduled to undergo planned awake intraoperative neurophysiology and single-neuronal recordings for deep brain stimulation targeting. Consideration for surgery was made by a multidisciplinary team including neurologists, neurosurgeons, and neuropsychologists. The decision to perform surgery was made independently of study candidacy or enrollment. Further, all microelectrode entry points and placements were based purely on planned clinical targeting and were made independently of any study consideration.<br><br>Once and only after a patient was consented and scheduled for surgery, their candidacy for participation in the study was reviewed with respect to the following inclusion criteria: 18 years of age or older, right-hand dominant, capacity to provide informed consent for study participation and demonstration of English fluency. All participants gave informed consent to participate in the study and were free to withdraw at any point without consequence to clinical care.

Ethics oversight	Massachusetts General Hospital Institutional Review Board

Note that full information on the approval of the study protocol must also be provided in the manuscript.
